# A new dimension in drug discovery: reversing epithelial–mesenchymal transition (EMT)

**DOI:** 10.1038/cddis.2016.316

**Published:** 2016-10-13

**Authors:** Ruby Yun-Ju Huang, Thomas Yo-Yan Huang

**Affiliations:** 1 Cancer Science Institute of Singapore, National University of Singapore, Singapore 117599, Singapore; 2 Department of Obstetrics & Gynaecology, National University Health System, Singapore 119228, Singapore; 3 Department of Anatomy, Yong Loo Lin School of Medicine, National University of Singapore, Singapore, Singapore; 4 National University Cancer Institute of Singapore, National University Health System, Singapore 119228, Singapore; 5 Department of Biology, University of California, San Diego, CA 92092, USA

Epithelial–mesenchymal transition (EMT) is a mechanism in which differentiated epithelial cells can lose their epithelial features. Some features include apicobasal polarity and cell adhesion system. Also, epithelial cells can acquire certain mesenchymal traits that are usually indicative of increased migratory and invasive properties.^1^ Stimuli, from the tumor microenvironment, induce carcinoma cell to undergo EMT. Subsequently, the carcinoma cells acquire chemoresistance and stem-cell properties.^2^ Downstream to these cues, the molecular machinery involves dynamic epigenetic, transcriptional, and post-translational control of the EMT effectors, such as the *E-cadherin* gene; those machinery plays part in maintaining the mesenchymal trait and executing the EMT.^1^ However, the gain of the mesenchymal trait is perhaps not the most dominant effect of EMT. Rather, the loss of the epithelial trait is more frequently observed. Therefore, to achieve mesenchymal–epithelial transition, which is the reversal of EMT, two approaches would be required to either abolish the mesenchymal or to restore the epithelial features.

In our recent publication,^3^ we described a promoter-reporter-based bioluminescent assay platform, which aimed to discover compounds that could restore the epithelial gene expression. This epithelial marker promoter induction (EpI) screen utilizes a sequence at the promoter region of the prototypic epithelial gene, *E-cadherin*, that is coupled in a firefly luciferase reporter system. The EpI screen was first performed in an ovarian cancer cell line with an intermediate EMT state. Later, the EpI screen was validated in other carcinoma cell lines with similar EMT states. We further demonstrated that promoter sequences of other epithelial markers, such as the epithelial-specific epidermal growth factor receptor family member *ERBB3*, can be incorporated into this EpI screen. Selecting from a compound library, we identified a class of compounds that manifests consistent and robust EpI effects, the histone deacetylase inhibitors (HDACi). Among the 41 HDACi screened, Mocetinostat was found to be the most potent compound that demonstrated the lowest concentration required for 50% of EpI (EpIC-50).

Currently, there is limited high-throughput screening (HTS) drug discovery platform designed for EMT. Contemporary platforms utilize EMT-related phenotypic functional assays, targeting the colony scattering or migration and invasion.^4, 5^ The philosophy of EMT reversal is to eliminate the mesenchymal cells that might have acquired therapeutic resistance and cancer stem-cell-like properties.^1^ Since EMT is a spectrum of intermediary states,^6^ it would be crucial to know at which threshold these aggressive features diminish. The reversal of EMT is not without consequences, because metastatic colonization is known to require mesenchymal–epithelial transition to occur.^7^ Therefore, it is important to carefully define and position the application of EMT reversal. In designing a readout assay for EMT, one practical and philosophical consideration is how much and how far should one reverse EMT. The key to success for assay development relies heavily on robust quantitative measurements of designed end point readouts with high reproducibility and tight sample variations. Any drug discovery assay for EMT would be required to fulfill these considerations.

Bioluminescent assays have been utilized for HTS in drug discovery.^8^ Because of their low backgrounds in the mammalian system, the dynamic linear range of theses assays can be very wide. The firefly and *Renilla* luciferases are the most versatile luciferases that act as genetic reporters in HTS. By incorporating the upstream gene regulatory elements (RE) with the luciferase gene, these assays can be associated with the regulation of gene transcription. The direct transcriptional control of EMT is achieved by the binding of EMT inducing transcription factors (EMT-TFs), such as the SNAIL and ZEB family, to their target epithelial genes.^1^ The SNAIL and ZEB family EMT-TFs act as transcriptional repressors and recognize the palindromic enhancer-box (E-box) DNA sequences, CANNTG, via their zinc-finger DNA binding domains.^9^ Therefore, the E-box at the promoter sites of epithelial genes is pivotal for EMT execution. This provides the rationale to utilize the E-box as the RE in developing luciferase reporter assay for EMT drug screening. Within the short 233 base pair (−108/+125) of *E-cadherin* promoter sequences, three E-boxes are present. In cells characterized by intermediate EMT states, the high expression of SNAIL and ZEB family EMT-TFs occupy these E-boxes and suppress the transcription, resulting in low luciferase activities. Therefore, compounds, that could ‘lift' these transcriptional suppressions at the E-box, would induce luciferase activities downstream. The dose-dependency of HDACi for EpI activities not only suggests that the transcriptional repression at the E-box is ‘lifted', but also indicates that there is dynamic linear control of the epigenetic and transcriptional regulation of epithelial differentiation. Thus, the derivation of EpIC-50 provides a useful tool, in a quantitative manner, to assess the degree of restoring epithelial differentiation. Moreover, this EpI platform can be applied to other epithelial differentiation genes. Grainyhead-like 2 (GRHL2) has been demonstrated as an EMT suppressor that forms a negative regulatory loop with ZEB1 and miR200 family.^10^ GRHL2 binds to the enhancer site at the second intron of the *E-cadherin* gene; furthermore, GRHL2 regulates its promoter activity via local DNA looping.^11^ Since GRHL2 is an important epithelial gatekeeper, GRHL2 target genes could be candidates for the EpI screen. For example, the gene encoding an epidermal growth factor receptor family member, *ERBB3*, could be considered as a candidate. Within the 1.2 kb length of *ERBB3* promoter, in addition to two E-boxes, there are two GRHL2 binding sites.^3^ As the direct transcriptional target for GRHL2, *ERBB3* expression would be downregulated during EMT, and GRHL2 is lost.^10^ Thus, *ERBB3*-EpI activity could be utilized as a secondary validation,^3^ and it could be applied at the initial phase of EpI screen.

One can envision the incorporation of various EpI reporters covering different REs to be essential for EMT drug discovery. EMT is a mechanism that converges the diverse upstream signaling pathways with dynamic control of various EMT effectors. Thus, EMT drug discovery approaches must evolve from existing pathway-centered paradigms. The functional difference between epithelial and mesenchymal cells provides the basis for phenotypic screens. The transcriptional regulation between EMT-TFs and EMT effectors further provides the basis for a screen-like EpI. Figure 1 summarizes the utilization of different epithelial specific REs (ERE) to constitute the basis of EpI screen. Hence, EMT drug discovery pipelines, which merges the phenotypic screens with EpI screens of a tumor microenvironment model, are required to find novel therapeutic agents. In pursuing precision medicine, pre-clinical models utilizing patient-derived materials have given us hope to real-time monitoring of therapeutic responses. During the clinical course of breast cancer patients,^12^ the identification of hybrid epithelial–mesenchymal clones of circulating tumor cells has given us a glimpse of future possibilities in the context of real-time monitoring of EMT. Patient-derived three-dimensional spheroid, or organoid cultures, would be a promising platform to incorporate phenotypic and EpI screens. However, one can expect that several technical hurdles, including capturing, preserving, and amplifying minute quantity of in-transit cells in culture conditions, would need to be overcome. It requires the incorporation of technology, such as droplet microfluidics,^13^ to encapsulate in-transit cells and to develop robust reporter assays at the single-cell level^14^ for downstream screening. Yet, recapitulating the microenvironment in this kind of system would be another challenge. The future of EMT drug discovery requires much creativity in combining multidisciplinary science at the forefront and to go beyond the binary view of EMT.^1^

A drug discovery screening-like EpI might not appeal to skeptics of the clinical relevance of EMT. Despite of such skepticism, the EpI screen strategy integrates our understandings of transcriptional regulation in epithelial differentiation and modifications of epithelial plasticity. Hence, we might be able to give new meanings to numerous leads that have been dropped from the traditional drug screening for cytotoxicity, and we could rescue them for further clinical development with revised positioning. It might sound naïve, but the EpI screen could be an effective strategy amidst the drug drought and patent cliff.

## Figures and Tables

**Figure 1:**
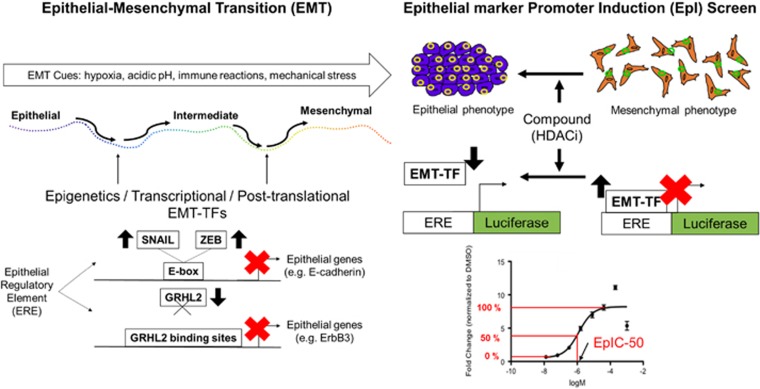
Rationale of the EpI screen. EMT can be triggered by multifarious cues such as hypoxia, acidic pH, immune reactions, and mechanical stress. There are three states in the EMT: epithelial, intermediate, and mesenchymal. When a cancer cell undergoes EMT, alterations may occur at the epigenetic, transcriptional, and post-translational levels. EpI screen searches for drugs that reverses the effects of EMT-TFs on epithelial regulator elements. When EMT-TFs, such as SNAIL and ZEB, are upregulated, they sit on E-boxes and decreases the expression of *E-cadherin*, an epithelial gene. In addition, when an EMT-TF, such as GRHL2, is downregulated and does not bind to its binding sites, epithelial genes such as *ERBB3* is suppressed Thus, a suppression of epithelial genes is an indicator of EMT, transforming the epithelial phenotype into a mesenchymal phenotype of a cancer cell. If the suppression of epithelial genes is removed, mesenchymal cells are able to regain its epithelial traits. In the drug discovery pipeline of EpI screen, the incorporation of epithelial regulator elements with luciferase, a bioluminescent enzyme, enables the quantification of potency of drugs in reversing EMT. A logarithmic concentration graph is plotted to determine the concentrations of drugs (logM) utilized from a library of HDACi to induce fold change, normalized to DMSO, in *E-cadherin* EpI activity. The arrow of EpIC-50 indicates the concentration corresponding to a 50% of maximum fold change
